# Comparative Studies of Vertebrate Platelet Glycoprotein 4 (CD36)

**DOI:** 10.3390/biom2030389

**Published:** 2012-09-24

**Authors:** Roger S. Holmes

**Affiliations:** Eskitis Institute for Cell and Molecular Therapies, Griffith University, Nathan, QLD 4111, Australia; Email: r.holmes@griffith.edu.au; Tel.: +61-7-3735-7773

**Keywords:** vertebrates, amino acid sequence, CD36, evolution, thrombospondin receptor

## Abstract

Platelet glycoprotein 4 (CD36) (or fatty acyl translocase [FAT], or scavenger receptor class B, member 3 [SCARB3]) is an essential cell surface and skeletal muscle outer mitochondrial membrane glycoprotein involved in multiple functions in the body. CD36 serves as a ligand receptor of thrombospondin, long chain fatty acids, oxidized low density lipoproteins (LDLs) and malaria-infected erythrocytes. CD36 also influences various diseases, including angiogenesis, thrombosis, atherosclerosis, malaria, diabetes, steatosis, dementia and obesity. Genetic deficiency of this protein results in significant changes in fatty acid and oxidized lipid uptake. Comparative CD36 amino acid sequences and structures and *CD36* gene locations were examined using data from several vertebrate genome projects. Vertebrate CD36 sequences shared 53–100% identity as compared with 29–32% sequence identities with other CD36-like superfamily members, SCARB1 and SCARB2. At least eight vertebrate CD36 *N*-glycosylation sites were conserved which are required for membrane integration. Sequence alignments, key amino acid residues and predicted secondary structures were also studied. Three CD36 domains were identified including cytoplasmic, transmembrane and exoplasmic sequences. Conserved sequences included *N*- and *C*-terminal transmembrane glycines; and exoplasmic cysteine disulphide residues; TSP-1 and PE binding sites, Thr92 and His242, respectively; 17 conserved proline and 14 glycine residues, which may participate in forming CD36 ‘short loops’; and basic amino acid residues, and may contribute to fatty acid and thrombospondin binding. Vertebrate *CD36* genes usually contained 12 coding exons. The human *CD36* gene contained transcription factor binding sites (including PPARG and PPARA) contributing to a high gene expression level (6.6 times average). Phylogenetic analyses examined the relationships and potential evolutionary origins of the vertebrate *CD36* gene with vertebrate *SCARB1* and *SCARB2* genes. These suggested that *CD36* originated in an ancestral genome and was subsequently duplicated to form three vertebrate *CD36* gene family members, *SCARB1*, *SCARB2* and *CD36*.

## 1. Introduction

Platelet glycoprotein 4 (CD36) (cluster of differentiation 36) (or fatty acyl translocase [FAT]; and scavenger receptor class B, member 3 [SCARB3]) is one of at least three members of the CD36-like family that is an integral membrane protein of many tissues of the body which plays a role in fatty acyl translocation and as a multiple ligand cell surface receptor of oxidized LDL lipoproteins (ox-LDL), long chain fatty acids, aged neutrophils and *Plasmodium falciparum*-parasitized erythrocytes (PE) which has been implicated in several diseases including insulin resistance, diabetes, atherosclerosis and malaria [1,2,3,4,5,6,7,8,9,10]. CD36 has also been reported on the outer mitochondrial membrane of skeletal muscle and serves a long chain fatty acid transport role, as well as contributing to the regulation of fatty acid oxidation by muscle mitochondria [11]. In addition, CD36 contributes to cerebrovascular oxidative stress and neurovascular dysfunction induced by amyloid-beta in Alzeheimer’s dementia [12,13] and may serve a ‘lipid-sensing’ role in the body with a broad physiological role as a lipid-receptor protein which influences eating behavior and energy balance [14]. Moreover, a specific CD36-dependent signaling pathway has been proposed for platelet activation by ox-LDL [15].

SCARB1 (also called CLA1, SRB1 and CD36L1), a second member of the CD36-like family, is a homo-oligomeric plasma membrane cell surface glycoprotein receptor for high density lipoprotein cholesterol (HDL), other phospholipid ligands and chylomicron remnants [16,17,18,19,20]. SCARB2 (also called LIMP2 (lysosomal integral membrane protein), SRB2 and CD36L2) is a third member of the CD36 family predominantly integrated within lysosomal and endosomal membranes which contributes to lysosomal membrane organization and transport functions [21,22,23,24,25].

The gene encoding CD36 (*CD36* in humans; *Cd36* in mice) is localized on chromosome 7 q11.2 and is encoded by 15 exons, including 12 coding exons [26,27,28,29]. Human *CD36* is expressed at very high levels in various cells and tissues of the body, including platelets, monocytes/macrophages, and microvascular endothelial cells, plays important roles in atherosclerosis, inflammation, thrombosis and angiogenesis [4,6,7,30,31,32], and is upregulated in human monocytes following statin administration [33]. Studies of *Cd36¯/Cd36¯* knockout mice have shown that *CD36*-deficiency protects against Western-type diet related cardiac dysfunction [34,35,36] and contributes to a reduction in fatty acid oxidation by muscle mitochondria [11,37]. Human clinical studies have also examined *CD36* polymorphisms associated with enhanced atherosclerotic cardiovascular diseases [38,39], type II diabetes [9], oral fat perception, fat preference and obesity in African-Americans [40] and protection from malaria [41,42]. In addition, hepatic *CD36* upregulation has been shown to be associated with insulin resistance, hyperinsulinaemia, and increased steatosis in patients with non-alcoholic steatohepatitis and chronic hepatitis C [43]. Reviews of the role of macrophage human CD36 in atherosclerosis have been published [7,44].

This paper reports the predicted gene structures and amino acid sequences for several vertebrate *CD36* genes and proteins, the secondary structures for vertebrate CD36 proteins, several potential sites for regulating human *CD36* gene expression and the structural, phylogenetic and evolutionary relationships for these genes and enzymes with those for vertebrate *CD36*, *SCARB1* and *SCARB2* gene families.

## 2. Results and Discussion

### 2.1. Alignments of Vertebrate CD36 Amino Acid Sequences

**Figure 1:**
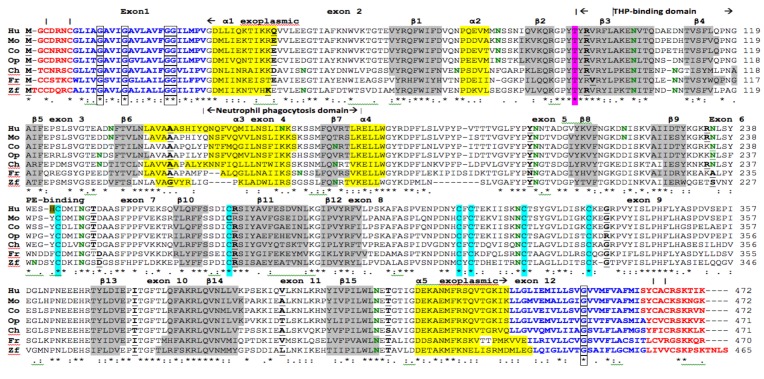
Amino Acid Sequence Alignments for Vertebrate CD36 Sequences. See Table 1 for sources of CD36 sequences; Hu-human; Mo-mouse; Co-cow; Op-opossum; Ch-chicken; Fr-frog; Zf-zebrafish; * shows identical residues for CD36 subunits; : similar alternate residues; . dissimilar alternate residues; predicted cytoplasmic residues are shown in 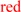; predicted transmembrane residues are shown in 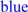; *N*-glycosylated and potential *N*-glycosylated Asn sites are in 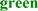; exoplasmic Thr92, which is phosphorylated by pyruvate kinase alpha, is shown in 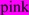; predicted disulfide bond Cys residues are shown in 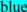; predicted α-helices for vertebrate CD36 are in 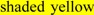 and numbered in sequence from the start of the predicted exoplasmic domain; predicted β-sheets are in 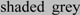 and also numbered in sequence; bold underlined font shows residues corresponding to known or predicted exon start sites; exon numbers refer to human *CD36* gene exons; 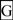 residues refer to conserved glycines in the *N*- and *C*-terminal oligomerisation domains of the trans-membrane sequence [49]; CD36 binding domains are identified: THP-refers to binding region for low-density lipoproteins [6,7,8]; neutrophil phagocytosis domain designated by [3,7]; PE binding refers to cytoadherence region of *Plasmodium falciparum*-parasitized erythrocytes (PE) to endothelial cells [4].

The deduced amino acid sequences for cow (*Bos taurus*), opossum (*Monodelphis domestica*), chicken (*Gallus gallus*), frog (*Xenopus tropicalis*) and zebrafish (*Danio rerio*) CD36 are shown in Figure 1 together with previously reported sequences for human and mouse CD36 (Table 1) [45,46]. Alignments of human with other vertebrate CD36 sequences examined were 53–100% identical, suggesting that these are products of the same family of genes, whereas comparisons of sequence identities of vertebrate CD36 proteins with human SCARB1 and SCARB2 proteins exhibited lower levels of sequence identities (30–32%), indicating that these are members of distinct *CD36*-like gene families (Supplementary Table 1)

The amino acid sequences for eutherian mammalian CD36 contained 472 residues, whereas opossum (*Monodelphis domestica*), platypus (*Ornithorhynchus anatinus*) and chicken (*Gallus gallus*) CD36 sequences contained 471 residues, while frog (*Xenopus tropicalis*) and zebrafish (*Danio rerio*) CD36 sequences contained 470 and 465 amino acids, respectively (Table 1; Figure 1). Previous studies have reported several key regions and residues for human and mouse CD36 proteins (human CD36 amino acid residues were identified in each case). These included cytoplasmic *N*-terminal and *C*-terminal residues: residues 2-6 and 462-472; *N*-terminal and *C*-terminal trans-membrane helical regions: residues 7-28 and 440-461 [32,45]; palmitoylated cysteine residues (Cys3; Cys7; Cys464; and Cys466) in the *N*- and *C*-terminal CD36 cytoplasmic tails [47]; exoplasmic Thr92, which is phosphorylated by protein kinase C alpha and contributes to the suppression of thrombospondin-1 binding *in vitro* [48]; His242 which contributes to the interaction of CD36-dependent endothelial cell adherence with *Plasmodium falcurum* [4]; and six exoplasmic disulfide bond forming residues: Cys243, Cys272, Cys311, Cys313, Cys322 and Cys333 [49].

**Table 1:** *CD36, SCARB1* and *SCARB2* genes and proteins. RefSeq: the reference amino acid sequence; ¹predicted Ensembl amino acid sequence; na-not available; GenBank IDs are derived from NCBI http://www.ncbi.nlm.nih.gov/genbank/; Ensembl ID was derived from Ensembl genome database http://www.ensembl.org; * designates scaffold; Un refers to unknown chromosome; UNIPROT refers to UniprotKB/Swiss-Prot IDs for individual CD36-like proteins (see http://kr.expasy.org); Un-refers to unknown chromosome; bps refers to base pairs of nucleotide sequences; the number of coding exons are listed; gene expression levels are in **bold**.

*CD36 Gene*	Species	RefSeq ID ¹Ensembl/NCBI	GenBank ID	UNIPROT ID	Amino acids	Chromosome location	Coding Exons (strand)	Gene Size bps	Gene Expression Level
**Human **	*Homo sapiens*	NM_001001547	BC008406	P16671	472	7:80,275,645-80,303,732	12 (+ve)	72,231	**6.6**
**Chimpanzee**	*Pan troglodytes*	¹XP_519573	na	na	472	7:81,142,402-81,169,764	12 (+ve)	#27,363	na
**Orangutan**	*Pongo abelii*	¹XP_002818343	na	na	472	7:95,750,733-95,779,630	12 (-ve)	#28,898	na
**Gibbon**	*Nomascus leucogenys*	¹XP_003252221	na	na	472	*GL397261:11,570,433-11,598,114	12 (+ve)	#27,682	na
**Rhesus**	*Macaca mulatta*	NP_001028085	na	na	472	3:136,626,102-136,653,066	12 (+ve)	#27,682	na
**Mouse**	*Mus musculus*	NM_001159555.1	BC010262	Q08857	472	5:17,291,543-17,334,712	12 (-ve)	43,170	**4.2**
**Rat**	*Rattus norvegicus*	NP_113749	L19658	Q07969	472	4:13,472,534-13,522,334	12 (+ve)	49,801	**0.3**
**Guinea Pig**	*Cavia porcellus*	¹XP_003469862	na	na	472	*31:20,074,611-20,098,210	12 (+ve)	#23,600	na
**Cow**	*Bos taurus*	NM_17410	BC103112	P26201	472	4:40,585,624-40,614,621	12 (-ve)	#28,998	na
**Dog**	*Canis familaris*	NM_001177734	ADE58431	na	472	18:23,334,171-23,360,045	12 (+ve)	#25,875	na
**Pig**	*Sus scrofa*	NP_001038087	AK400585	Q3HUX1	472	9:93,204,848-93,241,842	12 (-ve)	#36,995	na
**Rabbit**	*Oryctolagus cuniculus*	¹XP_002712062	na	na	472	7:35,303,111-35,333,630	12 (-ve)	#30,520	na
**Horse**	*Equus caballus*	¹XP_001487957	na	na	472	4:6730,96-698,607	12 (-ve)	#25,512	na
**Elephant**	*Loxodonta africana*	¹XP_003407226	na	na	472	5: 69,036,730-69,073,879	12 (-ve)	#37,150	na
**Opossum**	*Monodelphis domestica*	¹XP_001364375	na	na	471	8:149,041,138-149,075,533	12 (-ve)	#34,396	na
**Platypus**	*Ornithorhynchus anatinus*	¹XP_001506583	na	na	471	*Ultra5:3,505,963-3,536,963	12 (-ve)	#31,001	na
**Chicken**	*Gallus gallus*	¹ENSGALG8439	AJ719746	F1NER9	471	1:12,077,308-12,107,415	12 (-ve)	30,108	na
**Lizard**	*Anolis carolinensis*	¹XP_003221568	na	na	472	5:93,087,943-93,120,933	12 (-ve)	#32,991	na
**Frog**	*Xenopus tropicalis*	NP_001107151	na	na	470	*GL172681:663,550-679,762	12 (-ve)	#16,213	na
**Zebrafish**	*Danio rerio*	NP_001002363.1	BC076048	Q6DHC7	465	4:21,594,449-21,606,961	12 (-ve)	12,513	na
***SCARB1* Gene**	**Species**	**RefSeq ID ¹Ensembl/NCBI **	**GenBank ID**	**UNIPROT ID**	**Amino acids**	**Chromosome location**	**Coding Exons (strand)**	**Gene Size bps**	**Gene Expression Level**
**Human**	*Homo sapiens*	NM_00505	BC022087	Q8WVT0	509	12:125,267,232-125,348,266	12 (-ve)	81,035	**13.7**
**Mouse**	*Mus musculus*	NM_001205082.1	BC004656	Q61009	509	5:125,761,478-125,821,252	12 (-ve)	63,985	**5.1**
**Chicken**	*Gallus gallus*	¹XP_415106	na	na	503	15:4,543,054-4,558,954	12 (+ve)	15,901	na
**Zebrafish**	*Danio rerio*	NM_198121	BC044516	E7FB50	496	11:21,526,513-21,572,478	12 (-ve)	45,684	na
***SCARB2* Gene**									
**Human**	*Homo sapiens*	NM_005506	BT006939	Q53Y63	478	4:77,084,378-77,134,696	12 (-ve)	50,316	**3.2**
**Mouse**	*Mus musculus*	NM_007644	BC029073	O35114	478	5:92,875,330-92,934,334	12 (-ve)	59,005	**3.6**
**Chicken**	*Gallus gallus*	¹XP_42093.1	BX931548	na	481	4:51,411,268-51,429,620	12 (+ve)	18,353	na
**Zebrafish**	*Danio rerio*	NM_173259.1	BC162407	Q8JQR8	531	5: 63,942,096-63,955,449	13 (+ve)	13,354	na
***CD36* Gene**									
**Lancelet**	*Branchiostoma floridae*	¹XP_002609178.1	na	na	480	Un:534,334,234-534,343,082	12 (+ve)	8,849	na
**Sea squirt**	*Ciona intestinalis*	¹XP_002127015.1	na	na	523	09p:2,872,362-2,873,903	1 (-ve)	1,542	na
**Nematode**	*Caenorhabditis elegans*	NM_067224	na	Q9XTT3	534	III:12,453,609-12,456,726	8 (+ve)	3,118	**4.6**
**Fruit fly**	*Drosophila melanogaster*	NP_523859	na	na	520	2R:20,864,606-20,867,116	6 (-ve)	#2,511	na

### 2.2. Comparative Sequences for Vertebrate CD36 N-Glycosylation Sites

Ten exoplasmic *N*-glycosylation sites for human CD36 have been previously identified for this protein (Figure 1; Table 2) [50]. One of these sites (site 2) contained a proline residue at the second position and may not function as an *N*-glycosylation site due to proline-induced inaccessibility [51]. Eight of these sites were predominantly retained among the 19 vertebrate CD36 sequences examined (sites 4, 5, 10, 15, 19, 23 and 25) (Figure 1; Table 2). The sequence conservation observed for these residues among the vertebrate CD36 sequences examined suggests that they contribute significantly to the structure and function of vertebrate CD36 as a glycoprotein. The multiple *N*-glycosylation sites observed for vertebrate CD36 sequences suggest a role for *N*-proteoglycan residues exposed on the external surface of plasma membranes in the performance of CD36 functions in binding various lipid molecules, including long chain fatty acids. This is also supported by recent animal model studies examining the impacts of reduced *N*-glycosylation upon cardiac long chain fatty metabolism, which demonstrated a key role for *N*-glycosylation in the recruitment of CD36 into cardiac membranes [52]. 

### 2.3. Conserved Glycines in the N-Terminal Domain of the CD36 Trans-Membrane Sequence

The *N*-terminal region for vertebrate CD36 sequences (residues 1-29 for human CD36) contained cytoplasmic (residues 2-7) and trans-membrane (residues 8-29) motifs which underwent changes in amino acid sequence but retained predicted cytoplasmic and trans-membrane properties in each case, respectively (Figure 1). Vertebrate *N*-terminal trans-membrane sequences, in particular, were predominantly conserved, especially for CD36 Gly12, Gly16 and Gly24/Gly25 residues, which were observed among the vertebrate CD36 sequences examined (Figure 1). Site directed mutagenesis studies of the related human SCARB1 sequence have demonstrated key roles for *N*-terminus trans-membrane sequence glycine residues, by facilitating oligomerisation and selective lipid uptake by SCARB1 conserved glycine residues [53] and similar roles may apply to the conserved *C*-terminal domain CD36 glycine residues. A recent report has shown, however, that CD36 is capable of binding acetylated and oxidized low-density lipoproteins as a monomer, even though multiple homo- and hetero-protein interactions are formed in the plasma membrane [8]. A conserved glycine residue was also observed for the vertebrate *C*-terminal trans-membrane sequences (human CD36 Gly452) (Figure 1), however the role for this residue has not been investigated.

**Table 2:** Predicted *N*-glycosylation sites for CD36 sequences. Numbers refer to amino acids in the acid sequences, including *N*-asparagine; K-lysine; I-isoleucine; H-histidine; S-serine; T-threonine; Q-glutamine; D-aspartate; Y-tyrosine; and V-valine. Note that there are 25 potential sites identified for vertebrate CD36 and other CD36-like sequences, including 10 sites for human CD36 (see [49]). *N*-glycosylation sites were identified using the NetNGlyc 1.0 web server (http://www.cbs.dtu.dk/services/NetNGlyc/). Higher probability *N*-glycosylation sites are in **bold**.

Vertebrate	Species	Site 1	Site 2*	Site 3	Site 4	Site 5	Site 6	Site 7	Site 8	Site 9	Site 10	Site 11	Site12	Site 13
**CD36**														
**Human **	*Homo sapiens*				**79NSSN**	**102NVTQ**					**134NFTV**	**163NKSK**		
**Chimp**	*Pan troglodytes*				**79NSSN**	**102NVTQ**					**134NFTV**	**163NKSK**		
**Orangutan**	*Pongo abelii*				**79NSSN**	**102NVTQ**					**134NFTV**	**163NKSK**		
**Gibbon**	*Nomascus leucogenys*				**79NSSN**	**102NVTQ**					**134NFTV**	**163NKSK**		
**Rhesus**	*Macaca mulatta*				**79NSSN**	**102NITQ**					**134NFTV**	**163NKSK**		
**Marmoset**	*Callithrix jacchus*				**79NSSN**	**102NVTQ**					**134NFTV**			
**Mouse**	*Mus musculus*				**79NSSK**	**102NITQ**					**134NFTV**			
**Rat**	*Rattus norvegicus*				**79NSSK**	**102NITQ**					**134NFTV**			
**Guinea Pig**	*Cavia porcellus*				**79NSSN**	**102NVTQ**					**132NDTF**			**172NRTL**
**Cow**	*Bos taurus*				**79NSSK**	**102NITQ**								**172NRTL**
**Horse**	*Equus caballus*				**79NSSK**	**102NITH**	**109NHTV**				**134NDTF**			
**Dog**	*Canis familaris*				**79NSSK**	**102NITH**								**172NRTV**
**Pig**	*Sus scrofa*				**79NSSV**	**102NITQ**					**132NDTF**			
**Rabbit**	*Oryctolagus cuniculus*				**79NSSN**	**102NVTQ**					**132NDTF**			
**Elephant**	*Loxodonta africana*				**79NSSN**	**102NITQ**					**132NDTF**			
**Panda**	*Ailuropoda melanoleuca*				**79NSSA**	**102NITH**					**132NDTL**			
**Opossum**	*Monodelphis domestica*				**79NSTK**	**102NLTQ**					**131NDSF**			
**Platypus**	*Ornithorhynchus anatinus*				**79NNSK**	**102NITK**								
**Chicken**	*Gallus gallus*	**46NGTI**	72NPSD			**102NITE**	**108NGTI**				**131NDTI**			**171NRTV**
**Zebra finch**	*Taeniopygia guttata*	**46NGGT**	72NPSE			**102NVTE**	**108NGTI**				**131NDTL**			**171NRTV**
**Lizard**	*Anolis carolensis*	**46NGTI**			**79NGSQ**	**102NITH**					**131NDTF**			
**Frog**	*Xenopus tropicalis*					**101NITQ**	**107NNTV**						**162NSSL**	
**Zebrafish**	*Danio rerio*	**47NGTL**				**103NITF**	**109NNTV**							**168NRTV**
**Tetraodon**	*Tetraodon nigroviridis*				**77NGTT**	**100NVTY**	**105NDST**						**162NSSL**	
**Sea squirt**	*Ciona intestinalis*			**74NVTN**			**120NKTY**		**143NGSE**					
**Lancelet**	*Branchiostoma floridae*					**100NITF**	**106NGTV**	**122NMSF**		**129NDTF**				
**Fruit fly**	*Drosophila melanogaster*			**80NVTN**	**90NGSK**		**118NGTL**							
**Vertebrate**	**Species**	**Site 14**	**Site 15**	**Site 16**	**Site 17**	**Site 18**	**Site 19**	**Site 20**	**Site 21**	**Site 22**	**Site 23**	**Site 24**	**Site 25**	**No of**
**CD36**														**Sites**
**Human **	*Homo sapiens*		**205NNTA**		**220NISK**		**235NLSY**		**247NGTD**		**321NCTS**		**417NETG**	**10**
**Chimp**	*Pan troglodytes*		**205NNTA**		**220NISK**		**235NLSY**		**247NGTD**		**321NCTS**		**417NETG**	**10**
**Orangutan**	*Pongo abelii*		**205NNTA**				**235NLSY**		**247NGTD**		**321NCTS**		**417NETG**	**9**
**Gibbon**	*Nomascus leucogenys*		**205NNTA**				**235NLSY**		**247NGTD**		**321NCTS**		**417NETG**	**9**
**Rhesus**	*Macaca mulatta*		**205NNTA**				**235NLSY**		**247NGTD**		**321NCTS**		**417NETG**	**9**
**Marmoset**	*Callithrix jacchus*		**205NNTA**				**235NLSY**		**247NGTD**		**321NCTS**		**417NETG**	**9**
**Mouse**	*Mus musculus*		**205NDTV**		**220NISK**		**235NLSY**		**247NGTD**		**321NCTS**		**417NETG**	**9**
**Rat**	*Rattus norvegicus*		**205NNTV**		**220NISK**		**235NLSY**		**247NGTD**		**321NCTS**		**417NETG**	**9**
**Guinea Pig**	*Cavia porcellus*		**205NNTA**		**220NISK**		**235NLSY**		**247NGTD**		**321NCTS**		**417NETG**	**10**
**Cow**	*Bos taurus*		**205NNTA**				**235NLSY**		**247NGTD**		**321NCTS**		**417NETG**	**8**
**Horse**	*Equus caballus*		**205NNTV**		**220NISK**		**235NLSY**		**247NGTD**		**321NCTS**		**417NETG**	**10**
**Dog**	*Canis familaris*		**205NNTV**		**220NVSQ**		**235NLSY**		**247NGTD**		**321NCTS**		**417NETG**	**9**
**Pig**	*Sus scrofa*		**205NNTS**	**206NTSD**			**235NLSY**		**247NGTD**		**321NCTS**		**417NETG**	**9**
**Rabbit**	*Oryctolagus cuniculus*		**205NNTV**		**220NISK**		**235NLSY**		**247NGTD**		**321NCTS**		**417NETG**	**9**
**Elephant**	*Loxodonta africana*		**205NNTV**				**235NLSY**		**247NGTD**		**321NCTS**		**417NETG**	**8**
**Panda**	*Ailuropoda melanoleuca*		**208NNTA**				**238NLSY**		**250NGTD**		**324NCTS**		**420NETG**	**8**
**Opossum**	*Monodelphis domestica*		**204NNTV**				**234NLSF**		**246NGTD**		**320NCTS**		**416NETG**	**8**
**Platypus**	*Ornithorhynchus anatinus*		**204NNTA**				**234NLSY**		**246NGTD**		**320NCTS**		**416NETG**	**7**
**Chicken**	*Gallus gallus*		**204NGTS**				**234NLSY**		**246NGTD**		**320NCTL**		**416NETA**	**10**
**Zebra finch**	*Taeniopygia guttata*		**205NGTS**						**247NGTD**		**321NCTI**		**417NESA**	**9**
**Lizard**	*Anolis carolensis*		**205NETL**				**232NKSM**		**247NTGD**		**321NCTG**		**417NETA**	**9**
**Frog**	*Xenopus tropicalis*		**202NGTA**						**245NGTD**		**319NCTA**		**415NETA**	**7**
**Zebrafish**	*Danio rerio*		**194NGTV**					**229NDSY**	**237NGSD**		**311NCTL**		**406NETA**	**9**
**Tetraodon**	*Tetraodon nigroviridis*		**200NGTA**					**228NRTV**	**243NGTD**		**317NCTL**		**416NETA**	**9**
**Sea squirt**	*Ciona intestinalis*		**232NQSR**			**260NMSE**			**276NGTD**	**346NHTV**				**7**
**Lancelet**	*Branchiostoma floridae*	**182NDSL**	**211NGTD**						**255NGTD**			**333NISI**	**420NEST**	**9**
**Fruit fly**	*Drosophila melanogaster*		**223NGTS**									**347NVSL**		**5**

### 2.4. Conserved Vertebrate CD36 Cysteine Residues

Ten cysteine residues of the vertebrate CD36 sequences were conserved, including two within each of the *N*- (Cys3 and Cys7) and *C*-terminal (Cys464 and Cys466) cytoplasmic sequences, and six within the vertebrate exoplasmic sequences (Cys243; Cys272; Cys311; Cys313; Cys322; and Cys333) (Figure 1). The CD36 *N*- and *C*-terminal conserved cytoplasmic cysteine residues have been shown to be palmitoylated [47], which may contribute to protein-protein interactions, protein trafficking and membrane localization [54]. Comparative studies of vertebrate SCARB1 sequences have shown that *N*- and *C*-terminal cytoplasmic sequences lacked any conserved cysteine residues in this region [55]. The six conserved exoplasmic vertebrate CD36 cysteine residues participate in disulfide bridge formation for bovine CD36 (Cys243-Cys311; Cys272-Cys333; and Cys313-Cys322), resulting in a 1-3, 2-6 and 4-5 arrangement of the disulfide bridges [49]. In contrast, vertebrate SCARB1 exoplasmic sequences contain only four conserved cysteine residues forming disulfide bridges (Cys281; Cys321; Cys323; and Cys334); a fifth cysteine (Cys251) was not conserved among vertebrate SCARB1 sequences [55]; and a conserved sixth cysteine (not observed in the CD36 sequence) (human SCARB1 Cys384) which functions in lipid transfer activity [56,57].

### 2.5. Predicted Secondary Structures for Vertebrate CD36

Predicted secondary structures for vertebrate CD36 sequences were examined (Figure 1), particularly for the exoplasmic sequences. α-Helix and β-sheet structures were similar in each case, with a α-helix extending beyond the *N*-terminal and *C*-terminal trans-membrane regions, forming α1 and α7, respectively. A consistent sequence of predicted secondary structure was observed for each of the vertebrate CD36 sequences: *N*-terminal cytoplasmic sequence--*N*-terminal transmembrane sequence--α1--β1--α2--β2--β3--β4--β5--β6--α3--β7--α4--β8--β10--β11--β12--β13--β14--β15--α5--*C*-terminal trans-membrane sequence--*C*-terminal cytoplasmic sequence. Further description of the secondary and tertiary structures for CD36 must await the determination of the three dimensional structure for this protein, particularly for the exoplasmic region which directly binds oxidized LDL lipids and a wide range of other lipid-like structures, including long chain fatty acids [1,2,3,4,5,6,7,8,9,10].

### 2.6. Conserved Proline, Glycine and Charged Amino Acid Residues within the CD36 Exoplasmic Domain

**Supplementary Figure 1:**
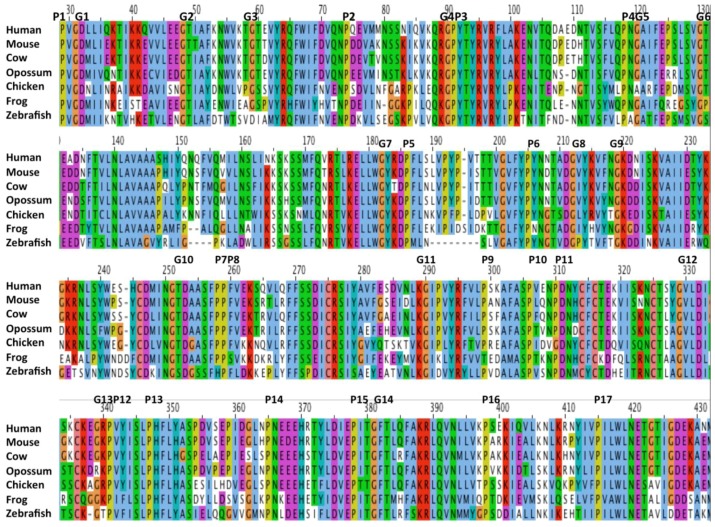
Amino Acid Sequence Alignments for Vertebrate CD36 Exoplasmic Sequences. Amino acids are color coded: yellow for proline (P); S (serine); green for hydrophilic amino acids, S (serine), Q (glutamine), N (asparagine), and T (threonine); brown for glycine (G); light blue for hydrophobic amino acids, L (leucine), I (isoleucine), V (valine), M (methionine), W (tryptophan); dark blue for amino acids, T (tyrosine) and H (histidine); purple for acidic amino acids, E (glutamate) and D (aspartate); and red for basic amino acids, K (lysine) and R (arginine); conserved prolines and glycines are designated as P1, P2 etc and G1, G2 etc, respectively. Numbers refer to human CD36 amino acid sequence.

Supplementary Figure 1 shows the alignment of 7 vertebrate CD36 amino acid sequences for the exoplasmic domain with colors depicting the properties of individual amino acids and conservation observed for some of these protein sequences. In addition to the key vertebrate CD36 amino acids detailed previously, others were also conserved, including 17 proline residues. A human CD36 genetic deficiency of one of these conserved prolines (Pro90→Ser) confirmed the significance of this residue, which lacked platelet CD36 [56]. Human CD36 deficiency has been shown to cause systemic metabolic changes in glucose and long chain fatty acid metabolism [59]. Prolines play a major role in protein folding and protein-protein interactions, involving the cyclic pyrrolidine amino acid side chain, which may introduce turns (or kinks) in the polypeptide chain as well as having destabilizing effects on α-helix and β-strand conformations [60]. In addition, the presence of sequential prolines within a protein sequence may confer further restriction in folding conformation and create a distinctive structure, such as that reported for the mammalian Na^+^/H^+^ exchanger, which plays a major role in cation transport [61]. Sequential prolines (Pro258-Pro259) were conserved for 6 of 7 vertebrate CD36 sequences examined and these may confer a distinctive conformation in this region supporting the lipid receptor functions for this protein. Moreover, regions of water exposed proteins with high levels of proline residues are often sites for protein-protein interactions [62] and these residues may significantly contribute to the binding of lipoproteins by the exoplasmic region of CD36. Similar results have been recently reported for the vertebrate SCARB1 exoplasmic region, however in this case, 30 conserved proline residues were observed [55].

Supplementary Figure 1 also shows conservation of 14 glycine residues for vertebrate CD36 exoplasmic domains, which due to their small size, may be essential for static turns, bends or close packing in the domain, or required for conformational dynamics during long chain fatty acid receptor on-off switching, as in the case of the aspartate receptor protein [63]. Both proline and glycine residues are frequently found in turn and loop structures of proteins, and usually influence short loop formation within proteins containing between 2 and 10 amino acids [61]. Evidence for these short loop structures within vertebrate CD36 exoplasmic sequences was evident from the predicted secondary structures for vertebrate CD36 (Figure 1), with proline and/or glycine residues found at the start of the following structures: α1 (Pro28; Gly30), β1 (Gly58), α2 (Pro73), β3 (Gly89-Pro90), β8 (Gly210), β12 (Gly287) and α5 (Gly420; Gly423). Moreover, CD36 sequential proline residues (Pro255-Pro256) were located in a region with no predicted secondary structure (between β9 and β10) but with disulfide bonds, which suggests that this is a region of conformational significance for CD36. 

In addition to the prolines and glycine residues for the vertebrate exoplasmic CD36 sequences, there are several conserved charged amino acid residue positions, including positively charged Lys40/Lys41 located within the first predicted exoplasmic helix (α1); Arg/Lys89, Arg95; Arg97 and Lys101 within or near the predicted strand-β3/strand β4 THP-binding domain region; Lys233/Lys235/Arg236 near the PE-binding domain; Lys263 located near the β10 strand; Arg276 within the β11 strand and adjacent to a disulphide bond; Lys288 which lies between predicted β11 and β12 strands; Lys337 and Arg/Lys340 near a disulphide bond; Lys388/Arg389 near the predicted β15 strand; and Lys401/Lys409 within the last exoplasmic helix (α5). Two domains of the exoplasmic CD36 sequence have been potentially implicated in the binding and endocytosis of apoptotic neutrophils: residues 155-183; and 93-120 (see [7]) The latter domain is called CLESH (for CD36 LIMP-II Emp [erythrocyte membrane protein] sequence homology) which is predominantly conserved, particularly near Thr92, which is phosphorylated by protein kinase C alpha and contributes to the suppression of thrombospondin-1 binding *in vitro* [48]. One or more of these positively charged CD36 exoplasmic regions may contribute to long chain fatty acid binding prior to the translocation of fatty acids inside the cell membrane. There are also several conserved acidic amino acid regions, particularly a sequence of three acidic amino acids (367Asp/68Asp/369Asp) near the β13 predicted strand. The conserved nature of these CD36 charged residues suggests that play key functional roles for this cell membrane protein, which may include serving as the long chain fatty acid CD36 receptor site. 

### 2.7. Alignments of Human CD36, SCARB1 and SCARB2

**Figure 2:**
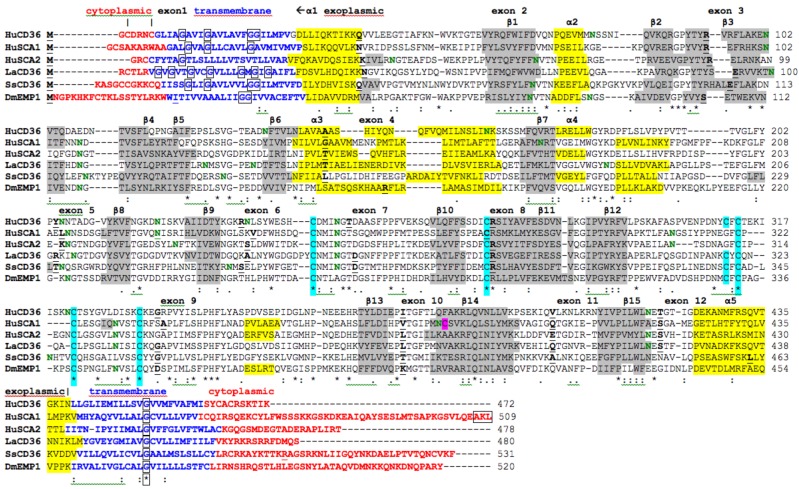
Amino Acid Sequence Alignments for Human CD36, SCARB1, and SCARB2; and Lancelet, Sea Squirt and Fruit Fly CD36-like Sequences. See Table 1 for sources of CD36-like sequences; HuCD36-human CD36; HuSCA1-human SCARB1; HuSCA2-human SCARB2; LaCD36-lancelet CD36; SsCD36- sea squirt CD36; DmEMP1-fruit fly endothelial membrane protein; * shows identical residues for subunits; : similar alternate residues; . dissimilar alternate residues; predicted cytoplasmic residues are shown in 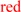; predicted trans-membrane residues are shown in 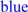; *N*-glycosylated and potential *N*-glycosylated Asn sites are shown in 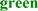; free-SH Cys involved in lipid transfer for human SCARB1 is shown in 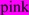; predicted disulfide bond Cys residues are shown in 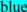; predicted α-helices for CD36-like sequences are in 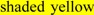 and numbered in sequence from the start of the predicted exoplasmic domain; predicted β-sheets are in 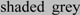 and also numbered in sequence; bold underlined font shows residues corresponding to known or predicted exon start sites; exon numbers are shown; 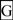 residues refer to conserved glycines in the *N*- and *C*-terminal oligomerisation domains of the trans-membrane sequence [49]; *C*-terminal SCARB1 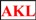 residues refer to PDZ-binding domain sequences [18,19].

The amino acid sequences for human CD36, SCARB1 and SCARB2 (see Table 1) are aligned in Figure 2. The sequences were 30-33% identical and showed similarities in several key features and residues, including cytoplasmic *N*-terminal and *C*-terminal residues; *N*-terminal and *C*-terminal trans-membrane helical regions; exoplasmic disulfide bond forming residues, previously identified for bovine CD36: Cys243-Cys311; Cys272-Cys333; and Cys313-Cys322 [47]; several predicted *N*-glycosylation sites for human CD36 (10 sites), SCARB1 (9 sites) and SCARB2 (9 sites), of which only two are shared between these sequences (*N*-glycosylation sites 15 and 21 (Table 2); and similar predicted secondary structures previously identified for SCARB1 [55] (Figure 1). The Cys384 residue, for which the free-SH group plays a major role in SCARB1-mediated lipid transport [57], was unique to SCARB1, being replaced by other residues for the corresponding CD36 and SCARB2 proteins (Phe383 and Ala379, respectively). *N*-terminal trans-membrane glycine residues, which play a role in the formation of SCARB1 oligomers [53], were also observed for the human CD36 sequence, with twin-glycines (Gly23-Gly24) conserved for the vertebrate CD36 sequences (Figure 1). In contrast, only one of these glycines (Gly10) was observed for the human SCARB2 sequence. These results suggest that human CD36, SCARB1 and SCARB2 proteins share several important properties, features and conserved residues, including being membrane-bound with cytoplasmic and transmembrane regions, having similar secondary structures, but being significantly different to serve distinct functions.

Alignments were also prepared for the predicted lancelet (*Branchiostoma floridae*) and sea squirt (*Ciona intestinalis*) CD36-like sequences and a major epithelial membrane protein (EMP) from fruit fly (*Drosophila melanogaster*) (FBpp0072309) with the human CD36, SCARB1 and SCARB2 sequences (Figure 2). The lancelet, sea squirt and fruit fly sequences examined shared many features with the CD36-like human sequences, including the *N*- and *C*-terminal cytoplasmic and transmembrane sequences; similarities in predicted secondary structures; positional identities for five conserved cysteine residues, indicating conservation of at least 2 disulfide bridges for these proteins; predicted *N*-glycosylation sites, including one which is shared across all 6 CD-like sequences (site 15 in Table 2); and trans-membrane glycine residues, which were observed in both the *N*- and *C*-terminal sequences.

### 2.8. Gene Locations and Exonic Structures for Vertebrate CD36 Genes

Table 1 summarizes the predicted locations for vertebrate *CD36* genes based upon BLAT interrogations of several vertebrate genomes using the reported human CD36 sequence [45] and the predicted sequences for other vertebrate *CD36* genes and the UC Santa Cruz genome browser [64]. Vertebrate *CD36* genes were transcribed on either the positive strand (e.g., human, chimpanzee, gibbon, rhesus, rat and dog genomes) or the negative strand (e.g., mouse, cow, pig, opossum, chicken, frog and zebrafish genomes). Figure 1 summarizes the predicted exonic start sites for human, mouse, cow, opossum, chicken, frog and zebrafish *CD36* genes with each having 12 coding exons, in identical or similar positions to those reported for the human *CD36* gene [28].

**Figure 3:**
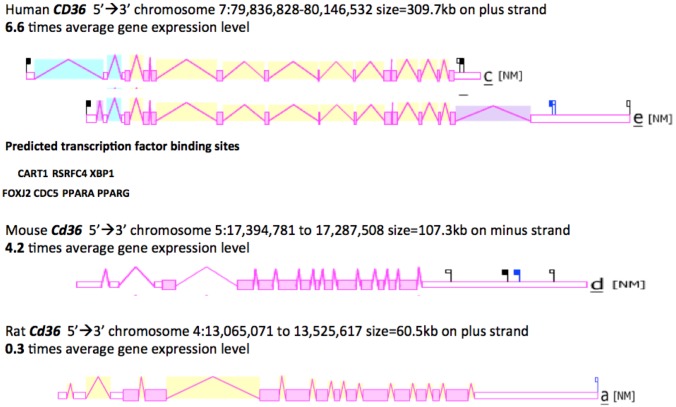
Gene Structures and Major Splicing Transcripts for the Human, Mouse and Rat *CD36* Genes. Derived from the AceView website http://www.ncbi.nlm.nih.gov/IEB/Research/Acembly/ mature isoform variants are shown with capped 5’- and 3’- ends for the predicted mRNA sequences [62]; NM refers to the NCBI reference sequence; exons are in pink; the directions for transcription are shown as 5’ → 3’; sizes of mRNA sequences are shown in kilobases (kb); predicted transcription factor binding sites (TFBS) for human *Cd36* are shown: CART1- a paired-class homeodomain transcription factor [71]; RSRFC4-myocyte enhancement factor 2A transcription factor [70]; XBP1-transcription factor [73]; FOXJ2-fork-head transcription factor [72]; CDC5-transcription activator and cell cycle regulator; [74]; PPARA-peroxisome proliferator-activated receptor alpha; and PPARG-peroxisome proliferator-activated receptor gamma [67,68].

Figure 3 shows the predicted structures of mRNAs for two major human *CD36* transcripts and the major *Cd36* transcripts for mouse and rat *Cd36* genes [46,65,66]. The human transcripts were ~2 kbs in length with 14 (isoform c) or 15 (isoform e) introns present for these *CD36* mRNA transcripts and in each case, a 3’-untranslated region (UTR) was observed. The human *CD36* genome sequence contained a number of predicted transcription factor binding sites (TFBS), including the dual promoter structure of PPARA (peroxisome proliferator-activated receptor-α) and PPARG (peroxisome proliferator-activated receptor-γ) sites [67,68]. Moreover, the mouse *Cd36* gene is regulated in a tissue specific manner by PPARA in liver and by PPARG in adipose tissues [69]. Other TFBS sites predicted for the human CD36 5’ promoter region included RSRFC4, a myocyte enhancer factor 2A found in muscle-specific and ‘immediate early’ genes [70]; CART1, a paired-class homeodomain transcription factor [71]; FOXJ2, a fork head transcriptional activator which is active during early development [72]; XBP1, a transcription factor which is critical for cell fate determination in response to endoplasmic reticulum stress [73]; and CDC5, a transcription activator and cell cycle regulator [74]. Hepatic upregulation of CD36 transcription in human patients has been recently shown to be significantly associated with insulin resistance, hyperinsulinaemia and increased steatosis in non-alcoholic steatohepatitis and chronic hepatitis C [43].

### 2.9. Comparative Human and Mouse CD36 Tissue Expression

**Figure 4:**
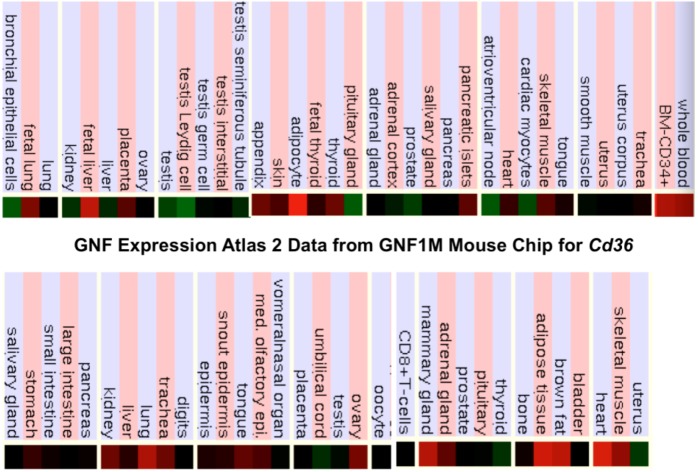
Comparative Tissue Expression for Human and Mouse *CD36* Genes. Expression ‘heat maps’ (GNF Expression Atlas 2 data) (http://biogps.gnf.org) were examined for comparative gene expression levels among human and mouse tissues for *CD36* genes showing high (red); intermediate (black); and low (green) expression levels [75]. Derived from human and mouse genome browsers (http://genome.ucsc.edu) [64].

Figure 4 presents ‘heat maps’ showing comparative gene expression for various human and mouse tissues obtained from GNF Expression Atlas Data using the U133A and GNF1H (human) and GNF1M (mouse) chips (http://genome.ucsc.edu; http://biogps.gnf.org) [75]. These data supported a broad and high level of tissue expression for human and mouse *CD36*, particularly for adipose tissue, heart, skeletal muscle and liver, which is consistent with previous reports for these genes [11,32,66]. Overall, human and mouse *CD36* tissue expressions levels were 4-6 times the average level of gene expression which supports the key role played by this enzyme in fatty acid metabolism, especially in liver, muscle and adipose tissue. 

The broad tissue and high level of gene expression reported for human and mouse *CD36* reflects key roles for this major cell membrane and muscle outer mitochondrial membrane glycoprotein in fatty acyl translocation and as a multiple ligand cell surface receptor of oxidized LDL lipoproteins (ox-LDL) and long chain fatty acids [7,11,33,66]. CD36 has also been described as a lipid ‘sensor’ playing a lipid receptor role for cells and tissues of the body [8,40]. Moreover, *CD36* upregulation is associated with insulin resistance and hyperinsulinaemia, leading to liver pathology and increased steatosis [43]. In addition, cardiomyocyte CD36 cell surface recruitment is induced by insulin, AMP-dependent protein kinase (AMPK) activity or contraction, and is regulated in its vesicular trafficking by the RabGAP-AS160 substrate and AS160-Rab8a GTPase activating protein (GAP) [76,77,78]. These features provide a link between cell membrane CD36 and the reported insulin-stimulated phosphorylation of AS160 involved with the translocation of the glucose transporter GLUT4 to the plasma membrane [79,80]. It is also relevant to report that plasma levels of soluble CD36 are increased in type 2 diabetic patients [81].

Significant levels of CD36 expression have also been described in brain tissues, where CD36 contributes to cerebrovascular oxidative stress and neurovascular dysfunction induced by amyloid-beta in Alzeheimer’s dementia [12,13], and in transporting long chain fatty acids across the blood-brain barrier [82].

### 2.10. Phylogeny of Vertebrate CD36-Like Sequences

**Figure 5:**
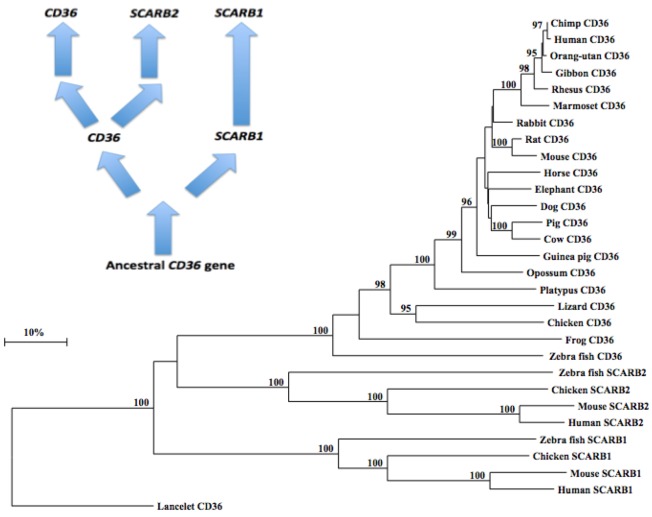
Phylogenetic Tree of Vertebrate CD36 Amino Acid Sequences with Human, Mouse, Chicken and Zebrafish SCARB1 and SCARB2 Sequences. The tree is labeled with the *CD36*-like gene name and the name of the animal and is ‘rooted’ with the lancelet CD36 sequence. Note the 3 major clusters corresponding to the *CD36*, *SCARB1* and *SCARB2* gene families. A genetic distance scale is shown. The number of times a clade (sequences common to a node or branch) occurred in the bootstrap replicates are shown. Only highly significant replicate values of 95 or more are shown with 100 bootstrap replicates performed in each case. A proposed sequence of *CD36* gene duplication events is shown.

A phylogenetic tree (Figure 5) was calculated by the progressive alignment of 21 vertebrate CD36 amino acid sequences with human, mouse, chicken and zebrafish SCARB1 and SCARB2 sequences. The phylogenetic tree was ‘rooted’ with the lancelet (*Branchiostoma floridae*) CD36 sequence (see Table 1). The phylogenetic tree showed clustering of the CD36 sequences into groups which were consistent with their evolutionary relatedness as well as groups for human, mouse, chicken and zebrafish SCARB1 and SCARB2 sequences, which were distinct from the lancelet CD36 sequence. These groups were significantly different from each other (with bootstrap values of ~100/100) with the clustering observed supporting a closer phylogenetic relationship between *CD36* and *SCARB2*, with the *SCARB1* gene being more distantly related. This is suggestive of a sequence of *CD36*-like gene duplication events: ancestral *CD36* gene duplication → *SCARB1* and *CD36* genes; followed by a further *CD36* duplication, generating the *SCARB2* and *CD36* genes found in all vertebrate species examined (Figure 5). It is apparent from this study of vertebrate *CD36*-like genes and proteins that this is an ancient protein for which a proposed common ancestor for the *CD36*, *SCARB1* and *SCARB2* genes may have predated the appearance of fish > 500 million years ago [83]. In parallel with the evolution of CD36 and other CD36-like proteins (SCARB1 and SCARB2), thrombospondins (TSPs) are also undergoing evolutionary changes in their structures and functions [84], with gene duplication events proposed at the origin of deuterostomes.

## 3. Methods

### 3.1. Vertebrate CD36 Gene and Protein Identification

BLAST (Basic Local Alignment Search Tool) studies were undertaken using web tools from the National Center for Biotechnology Information (NCBI) (http://blast.ncbi.nlm.nih.gov/Blast.cgi) [85]. Protein BLAST analyses used vertebrate CD36 amino acid sequences previously described [8,45] (Table 1). Non-redundant protein sequence databases for several vertebrate genomes were examined using the blastp algorithm from sources previously described [55]. This procedure produced multiple BLAST ‘hits’ for each of the protein databases which were individually examined and retained in FASTA format, and a record kept of the sequences for predicted mRNAs and encoded CD36-like proteins. Predicted CD36-like protein sequences were obtained in each case and subjected to analyses of predicted protein and gene structures. 

BLAT (Blast-like Alignment Tool) analyses were subsequently undertaken for each of the predicted CD36 amino acid sequences using the UC Santa Cruz Genome Browser [64] with the default settings to obtain the predicted locations for each of the vertebrate *CD36* genes, including predicted exon boundary locations and gene sizes. BLAT analyses were similarly undertaken for vertebrate *SCARB1* and *SCARB2* genes using previously reported sequences in each case (see Table 1). Structures for human and mouse isoforms (splicing variants) for human *CD36*, mouse *Cd36* and rat *Cd36* were obtained using the AceView website to examine predicted gene and protein structures [66]. 

### 3.2. Predicted Structures and Properties of Vertebrate CD36

Predicted secondary structures for vertebrate CD36 proteins, human SCARB1 and SCARB2, lancelet (*Branchiostoma floridae*) CD36, sea squirt (*Ciona intestinalis*) CD36 and a fruit fly (*Drosophila melanogaster*) epithelial membrane protein (FBpp0072309) were obtained using the PSIPRED v2.5 web site tools provided by Brunel University [86]. Molecular weights, *N*-glycosylation sites [49] and predicted trans-membrane, cytosolic and exocellular sequences for vertebrate SCARB1 proteins were obtained using Expasy web tools (http://au.expasy.org/tools/pi_tool.html). 

### 3.3. Comparative Human and Mouse CD36 Gene Expression

The genome browser (http://genome.ucsc.edu) [62] was used to examine GNF Expression Atlas 2 data using various expression chips for human and mouse *CD36* genes (http://biogps.gnf.org) [74]. Gene array expression ‘heat maps’ were examined for comparative gene expression levels among human and mouse tissues showing high (red); intermediate (black); and low (green) expression levels.

### 3.4. Phylogeny Studies and Sequence Divergence

Alignments of vertebrate CD36, SCARB1 and SCARB2 sequences were assembled using BioEdit v.5.0.1 and the default settings [87]. Alignment ambiguous regions, including the amino and carboxyl termini, were excluded prior to phylogenetic analysis yielding alignments of 431 residues for comparisons of vertebrate CD36 sequences with human, mouse, chicken and zebra-fish SCARB1 and SCARB2 sequences with the lancelet (*Branchiostoma floridae*) CD36 sequence (Table 1). Evolutionary distances and phylogenetic trees were calculated as previously described [85]. Tree topology was reexamined by the boot-strap method (100 bootstraps were applied) of resampling and only values that were highly significant (≥95) are shown [88]. 

## 4. Conclusions

The results of this study indicate that vertebrate *CD36* genes and encoded proteins represent a distinct gene and protein family of *CD36*-like proteins which share key conserved sequences that have been reported for other CD36-like proteins (SCARB1 and SCARB2) previously studied [16,17,18,19,20,21,22,23,24]. CD36 has a unique property among these proteins in serving a major role in fatty acyl translocation and as a multiple ligand cell surface receptor of oxidized LDL lipoproteins (ox-LDL), long chain fatty acids, aged neutrophils and *Plasmodium falciparum*-parasitized erythrocytes [3,4,5,6,7,8,9,10]. *CD36* is encoded by a single gene among the vertebrate genomes studied and is highly expressed in human and mouse tissues, particularly in adipose tissue, heart, skeletal muscle and liver, and usually contain 12 coding exons. Predicted secondary structures for vertebrate CD36 proteins showed strong similarities with other CD36-like proteins, SCARB1 and SCARB2. Three major structural domains were observed for vertebrate CD36 sequences, including *N*- and *C*-terminal cytoplasmic domains; *N*- and *C*-terminal trans-membrane domains; and an exoplasmic domain, which serves as the ‘receptor’ for long chain fatty acids and thrombospondins [5,6,7,8,14,32]. The latter domain contained three disulfide bridges [49]; several *N*-glycosylation sites for glycan binding (7–10 sites), which are essential for membrane recruitment [52]; 17 conserved proline and 14 glycine residues, which may contribute to short loop structures for the CD36 exoplasmic structure; and several conserved basic amino acid sites, which may promote long chain fatty acid binding. Phylogenetic studies using 21 vertebrate CD36 sequences with human, mouse, chicken and zebrafish SCARB1 and SCARB2 sequences indicated that the *CD36* gene appeared early in evolution, prior to the appearance of bony fish more that 500 million years ago, and has undergone at least two gene duplication events: ancestral *CD36* → vertebrate *SCARB1* and *CD36*; with the latter gene undergoing a further gene duplication generating vertebrate *CD36* and *SCARB2* genes.

## Supplementary

**Supplementary Table 1:** CD36, SCARB1 and SCARB2 proteins: subunit MWs and percentage identities. High % identities are in **bold**.

*CD36 Gene*	Species	Subunit MW	% Identity with human	% Identity with human	% Identity with human
			SCARB1	SCARB2	CD36
**Human **	*Homo sapiens*	53,053	31	30	**100**
**Chimpanzee**	*Pan troglodytes*	53,064	31	30	**100**
**Orangutan**	*Pongo abelii*	53,039	32	30	**97**
**Gibbon**	*Nomascus leucogenys*	53,161	32	30	**96**
**Rhesus**	*Macaca mulatta*	53,041	32	31	**94**
**Mouse**	*Mus musculus*	52,698	30	31	**83**
**Rat**	*Rattus norvegicus*	52,731	31	30	**86**
**Guinea Pig**	*Cavia porcellus*	53,085	32	32	**81**
**Cow**	*Bos taurus*	52,940	32	30	**82**
**Dog**	*Canis familaris*	52,549	31	30	**82**
**Pig**	*Sus scrofa*	53,085	31	30	**82**
**Rabbit**	*Oryctolagus cuniculus*	52,729	31	31	**88**
**Horse**	*Equus caballus*	52,789	31	31	**83**
**Elephant**	*Loxodonta africana*	52,873	31	31	**80**
**Opossum**	*Monodelphis domestica*	53,017	30	30	**73**
**Platypus**	*Ornithorhynchus anatinus*	52,807	31	30	**73**
**Chicken**	*Gallus gallus*	52,624	30	32	**61**
**Lizard**	*Anolis carolinensis*	52,890	31	31	**61**
**Frog**	*Xenopus tropicalis*	52,696	30	29	**55**
**Zebrafish**	*Danio rerio*	51,590	31	31	**53**
***SCARB1* Gene**	**Species**	**Subunit MW**	**% Identity with human**	**% Identity with human**	**% Identity with human**
			**SCARB1**	**SCARB2**	**CD36**
**Human**	*Homo sapiens*	56,973	**100**	29	31
**Mouse**	*Mus musculus*	56,754	**79**	29	29
**Chicken**	*Gallus gallus*	55,918	**57**	28	31
**Zebrafish**	*Danio rerio*	55,742	**51**	28	30
***SCARB2* Gene**					
**Human**	*Homo sapiens*	54,290	29	**100**	30
**Mouse**	*Mus musculus*	54,044	29	**85**	31
**Chicken**	*Gallus gallus*	53,907	30	**59**	33
**Zebrafish**	*Danio rerio*	60,234	31	**43**	33
***CD36* Gene**					
**Lancelet**	*Branchiostoma floridae*	54,141	34	35	35
**Sea squirt**	*Ciona intestinalis*	58,009	26	33	31
**Nematode**	*Caenorhabditis elegans*	60,182	21	26	24
**Fruit fly**	*Drosophila melanogaster*	58,663	20	23	26
